# “My Hand Is Different”: Altered Body Perception in Stroke Survivors with Chronic Pain

**DOI:** 10.3390/brainsci12101331

**Published:** 2022-09-30

**Authors:** Brendon S. Haslam, David S. Butler, G. Lorimer Moseley, Anthony S. Kim, Leeanne M. Carey

**Affiliations:** 1 Neurorehabilitation and Recovery, Florey Institute of Neuroscience and Mental Health, University of Melbourne, Melbourne 3010, Australia; 2 Occupational Therapy, School of Allied Health, Human Services and Sport, La Trobe University, Melbourne 3086, Australia; 3 IMPACT in Health, University of South Australia, Kaurna Country, Adelaide 5001, Australia; 4 Neuro-Orthopaedic Institute, Adelaide 5001, Australia; 5 Weil Institute of Neurosciences, Department of Neurology, University of California, San Francisco, CA 94143, USA

**Keywords:** pain, stroke, chronic pain, body perception disturbance, body image

## Abstract

Background: Chronic pain and body perception disturbance are common following stroke. It is possible that an interaction exists between pain and body perception disturbance, and that a change in one may influence the other. We therefore investigated the presence of body perception disturbance in individuals with stroke, aiming to determine if a perceived change in hand size contralateral to the stroke lesion is more common in those with chronic pain than in those without. Methods: Stroke survivors (N = 523) completed an online survey that included: stroke details, pain features, and any difference in perceived hand size post-stroke. Results: Individuals with stroke who experienced chronic pain were almost three times as likely as those without chronic pain to perceive their hand as now being a different size (OR = 2.895; 95%CI 1.844, 4.547). Further, those with chronic pain whose pain included the hand were almost twice as likely to perceive altered hand size than those whose pain did not include the hand (OR = 1.862; 95%CI 1.170, 2.962). This was not influenced by hemisphere of lesion (*p* = 0.190). Conclusions: The results point to a new characteristic of chronic pain in stroke, raising the possibility of body perception disturbance being a rehabilitation target to improve function and pain-related outcomes for stroke survivors.

## 1. Introduction

Stroke is a leading cause of disability worldwide [1]. Survivors of stroke commonly experience difficulties in mobility, in performing the activities of daily living, in speech and in mood [1,2]. Individuals with stroke also experience higher rates of persistent, or chronic pain [3,4] than is reported for the general (non-stroke) population [5,6]. People with stroke who also experience chronic pain have further difficulties in cognitive function and physical activity, and higher rates of fatigue, anxiety and depression [7,8], than people with stroke who do not experience chronic pain.

The pain experience following stroke is varied. Shoulder pain, headache, back pain and other limb joint pain are frequently experienced [4]. Survivors of stroke may have neuropathic characteristics to their pain, leading to diagnoses of conditions such as complex regional pain syndrome (CRPS) and, less frequently, central post-stroke pain [4,9]. Many survivors of stroke often experience novel pain (i.e., pain not previously experienced before the stroke) [10], commonly in the sub-acute and chronic phases [11]. The onset of pain during these phases is consistent with the development of pain post-stroke over time [4,12], and suggests that post-stroke pain often reflects a ‘mixed pain’, with nociceptive, neuropathic and nociplastic (i.e., due to adaptive processes) components as defined by the International Association for the Study of Pain [13]. Evidence of effective treatments for chronic pain post-stroke are limited, as reflected in the current stroke guidelines of Australia [14], Canada [15], the United Kingdom [16] and the United States of America [17], which often omit recommendations for management of pain, other than weak recommendations for post-stroke shoulder pain. Development of effective treatments based on robust evidence is required [18]. For this to occur, improved understanding of the contributions towards post-stroke pain, and potential mechanisms involved, is needed.

Disturbances in body perception have been described for a range of other challenging and complex pain states, including complex regional pain syndrome (CRPS) [19,20], fibromyalgia [21], chronic back pain [22,23] and chronic neck pain [24]. Body perception can be considered as the ‘experienced physical self’, or the ‘conscious experience of how one’s body feels to its owner’ [25]. The construction and ongoing maintenance of body perception is considered to be formed by tactile, proprioceptive and visual inputs, and modulated by memories, beliefs, attitudes and perceptions [26]. Supported by neuroimaging studies, it can be considered, that different neural networks are involved in how an individual perceives their body, with motor information providing ongoing knowledge related to body schema (i.e., body shape and postures) and sensory information related to body representation [27,28]. Body perception is malleable, as exemplified by the learning of ‘impossible movements’ of a phantom limb coinciding with the emergence of equally impossible configurations of limb-specific body perception [29]. Alterations in body perception may occur when the coping strategies of the individual related to body reality are overwhelmed by factors such as injury, disease, disability of social stigma [30]. It is also therefore plausible that it is associated with nociplastic, previously central sensitization, processing in the brain [31].

Body perception disturbance refers to an alteration in the size, shape or position of the experienced physical self [32]. Treatments that target reductions in body perception disturbance in people with chronic pain have shown preliminary success in reducing pain and increasing function [33,34,35]. However, while there is encouraging evidence that strategies targeting body perception may influence pain, most studies have failed to include a relevant assessment of body perception, the exception being a study showing improved body perception following mirror therapy for CRPS [36]. It remains possible that an interaction exists between pain and body perception disturbance, and that a change in one may influence the other.

Individuals with stroke are more likely to report body perception disturbance [37,38] than non-stroke individuals. This may in part be due to survivors of stroke often experiencing difficulties in performing active movements related to daily functional tasks [39], and somatosensory dysfunction related to tactile discrimination and proprioception [40], which are significant contributors to the construction and ongoing maintenance of body perception [26]. Presence of somatosensory dysfunction in the acute phase post-stroke is considered to be related to the infarct and interruption to specific brain regions and networks. However, it has also been proposed that there may be different factors beyond infarct lesion location that may contribute to the ongoing presence of somatosensory symptoms in individuals with stroke beyond 12 months [41]. It is currently not known whether a further relationship of altered body perception exists in individuals with stroke who experience chronic pain.

### Aims of This Study

The primary aim of the current study was to determine if individuals with sub-acute and chronic stroke (i.e., stroke > three months) who experience chronic pain were more likely to report changes in body perception (as indicated by presence of alterations in perceived hand size) than stroke survivors without pain. Second, if so, is this related to the pain affected region? If a relationship does exist, there may be potential in subsequent development of novel treatment interventions targeting body perception, and potentially pain, in individuals with stroke.

## 2. Materials and Methods

### 2.1. Study Design and Participants

A cross-sectional online observational study was developed for individuals who had experienced one or more strokes. The study utilized a survey, which was developed in consultation with: survivors of stroke; clinicians experienced in stroke rehabilitation; and researchers with stroke and/or chronic pain experience. The survey sought demographic data, medical history and stroke-related data, perceived hand-size data (see below), and data from selected pain scales (Numerical Rating Scale for Pain; Neuropathic Pain Symptom Inventory [42]) as appropriate. All responses were de-identified. Prior to commencement, pilot trials were performed by individuals with and without stroke, and survey completion was found to take 15–20 min to complete. The study protocol was approved by the Human Research Ethics Committee of the University of Melbourne, the Human Ethics Committee of La Trobe University, and the Institutional Review Board of the University of California, San Francisco. Data were collected between October 2015 and October 2018. This manuscript conforms to STROBE (Strengthening the Reporting of Observational Studies in Epidemiology) guidelines [43].

The study was publicized through various means: flyers, newsletters and website listings of agreeing stroke-related organizations, social media links and a research register for survivors of stroke. Potential participants were presented with an online project information sheet and asked if they wished to proceed. Upon agreeing, they were then directed to an online consent form, and once providing consent, were advanced to the online survey page. Participants were required to have English language skills, adequate computer skills and internet access. Individuals were included in the study if they were able to provide consent, were eighteen years or over, and had experienced their stroke at least three months prior, consistent with sub-acute or chronic phases post-stroke [44]. Individuals were excluded if they reported a diagnosis of any other neurological conditions, due to the possibility that this may impact independently on body perception [20]. All participants were asked to indicate if they experienced persistent pain over the past three months (yes/no)? This variable was used to allocate participants to groups (chronic pain or no chronic pain for the primary analysis). Those with reported chronic pain were requested to complete the Numerical Rating Scale for Pain and the Neuropathic Pain Symptom Inventory [42], and complete body charts to indicate any regions where pain was experienced. The presence of pain that included the hand was used to allocate participants with chronic pain into groups for the secondary analysis (pain including the hand or pain excluding the hand). All participants were asked about perceived hand size. A copy of the survey questions described has been included (Appendix A).

### 2.2. Instruments

#### 2.2.1. Numerical Rating Scale for Pain (NRS)

Participants who reported experiencing pain for greater than three months (i.e., chronic pain) indicated the average severity of their pain in response to the survey request “Please score the average severity of your persistent pain level out of 10 on the chart below, where 0 = no pain, and 10 = worst pain imaginable”, and were provided with an 11 point NRS anchored at left with “0 No pain”, and at right with “10 Worst pain imaginable”. The NRS has previously been used in large online studies [6] and has been shown to be valid and reliable with good sensitivity [45].

#### 2.2.2. Neuropathic Pain Symptom Inventory (NPSI)

Following completion of the NRS, participants who reported experiencing chronic pain were presented with an online copy of the Neuropathic Pain Symptom Inventory [42] and requested to respond as follows: “We would now like you to complete the Neuropathic Pain Symptom Inventory. In this, you will be asked some questions about the types of pain that you feel”. The NPSI is an assessment designed specifically for conditions such as stroke where neuropathic pain characteristics are likely [42]. The NPSI contains ten items that describe potential pain symptoms often experienced by people with neuropathic pain characteristics. Participants were requested “Please indicate the number that best describes the average severity of your (pain symptom) during the past 24 h. Choose the number 0 if you have not felt such pain. For each of the ten pain symptoms listed in the NPSI, an 11-point NRS anchored at left with “0 No (pain symptom)” and at right with “10 Worst (pain symptom) imaginable” was displayed in the same format as the NRS for Pain described above, consistent with the format of the NPSI [42]. The ratings for the ten pain symptoms are combined and then used to provide a total score out of 100 but are also grouped into five domains: superficial spontaneous burning pain; deep spontaneous pain; paroxysmal pain; evoked pain; and paresthesia/dysaesthesia. The items in each of these domains are averaged to provide domain-specific sub scores out of ten. The NPSI has been validated for use in individuals with neuropathic pain conditions such as stroke [42]. The online Australian/English version of the NPSI was used with the permission of the Mapi Research Trust, Lyon, France (www.proqolid.org, permission received 28 August 2013).

#### 2.2.3. Perceived Hand Size Question

All participants were provided with the question “Since your stroke, does it feel like your hand is now a different size?” and to indicate either “yes” or “no”. If the participant responded “yes”, the survey then asked the participant if it now felt bigger or smaller.

### 2.3. Data Analysis

Following application of the exclusion criteria, participants were allocated into groups for the primary analysis according to the presence or not of chronic pain and were excluded if missing data made grouping impossible. To test the hypothesis that there is an association between presence of chronic pain and changes in body perception, i.e., that perceived hand size differs across stroke groups with and without pain, the chi-square test was used, with factor ‘Group’. Odds ratios were generated to determine the strength of the association if present. The mean age of participants and chronicity of stroke in years were calculated for each group and compared using the Student t-test, while comparisons of lesion side were performed using the chi-square test. Secondary analyses performed using the chi-square test investigated differences in those with chronic pain according to region of pain experienced (i.e., pain including or excluding the hand; factor ‘Hand’) and odds ratios were again generated. Due to multiple comparisons between-group differences were deemed significant at *p* < 0.025. Finally, reported severity of pain for the NRS and the NPSI (total score and domain sub-scores) were compared using the Student t-test, with significance set at *p* < 0.05.

## 3. Results

**Figure 1:**
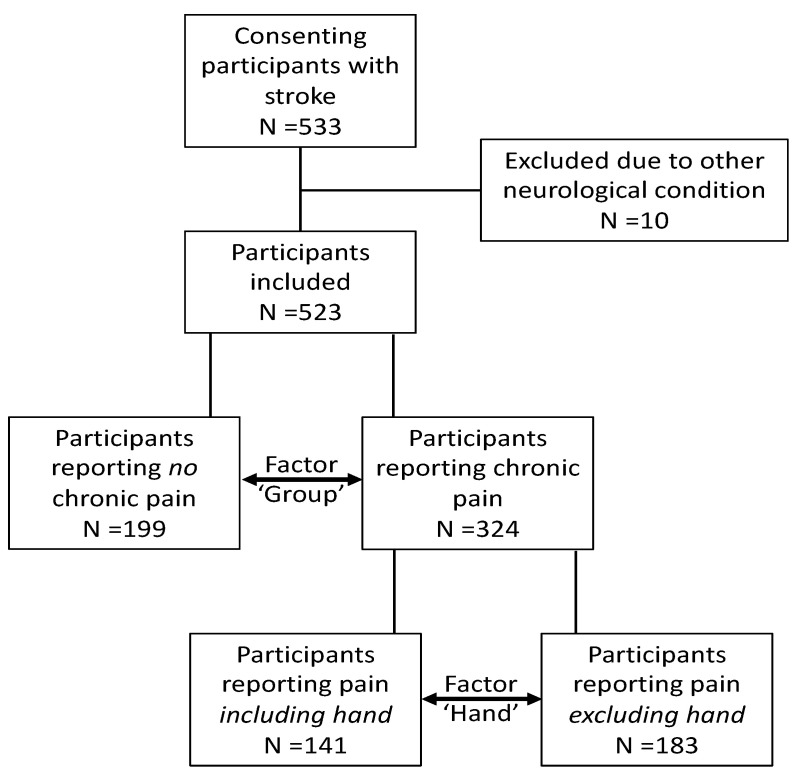
Flow of participants through the study.

A total of 533 individuals with stroke participated in the study (Figure 1). Ten participants were excluded because of other neurological conditions. Final data analysis was carried out on 523 participants (199 with no pain; 324 with chronic pain, of which 183 experienced chronic pain excluding the hand, and 141 experienced chronic pain including the hand). Power was calculated for the achieved sample size to observe a small-medium effect (0.2) in presence of altered perceived hand size between those with and without pain at a significance level of 0.025 and was calculated to be 0.990.

The mean age and duration post-stroke for each group, frequency of reported altered perceived hand size, hemispheric side of lesion and gender distribution are reported in Table 1. Ten participants chose not to indicate that they identified as being either female or male. Seventy-one of participants did not indicate the side of their stroke, selecting “unknown” or choosing to leave it blank, while eight participants who indicated that their hand now felt a different size failed to indicate whether it was either “bigger” or “smaller”. Thirty-seven participants reported that they had been diagnosed as having CRPS of the hand, representing 27% of those with chronic hand pain post-stroke.

No significant differences were found between the groups in age, reported side of lesion or duration post-stroke. Females were more likely than males to report chronic pain. Individuals with chronic pain were almost three times as likely to experience that their hand felt a different size following their stroke, than those without chronic pain (OR = 2.895; 95%CI 1.844, 4.547). Nominated gender did not influence likelihood of reporting alterations in perceived hand size (OR = −0.031; 95%CI 0.656, 1.433). For those stroke survivors who reported that their hand felt a different size following their stroke, the presence of chronic pain did not influence whether their hand was perceived to be bigger or smaller than it felt prior to their stroke. Comparison of perceived altered hand size by hemisphere of lesion did not show a significant difference between those with left (N = 181) and right (N = 238) hemisphere strokes (*p* = 0.190, calculated using the chi-square test).

Comparisons made between chronic pain groups based on presence of hand pain or not are presented in Table 2. Having chronic hand pain was associated with higher frequencies of perceived increased size of one’s hand, than chronic pain excluding the hand, while no significant differences were detected in frequency of decreased perceived size. Of those experiencing chronic pain, participants whose pain included their hand, were almost twice as likely to perceive altered hand size than those whose pain excluded the hand (OR = 1.862; 95%CI 2.962, 1.170). The reported side of the lesion was associated with significant differences in the frequencies of experienced pain by region (i.e., pain including or excluding the hand).

Of the stroke survivors who experienced chronic pain, no significant difference was detected in self-reported pain intensity as assessed using the NRS, between those whose pain included the hand and those whose pain did not. In contrast, those with hand pain demonstrated higher severity of neuropathic pain symptoms, which applied across all domains except paroxysmal stabbing and pressure evoked pain, as indicated in Table 3.

## 4. Discussion

Our results support the hypothesis that individuals with sub-acute and chronic stroke who experience chronic pain are more likely to report changes in body perception (as indicated by presence of alterations in perceived hand size) than those without pain. Further, the frequency of altered body perception of the hand, and strength of the association were greater when the region included the hand. This finding of altered body percept in a stroke population with chronic pain is consistent with other chronic pain populations such as knee osteoarthritis (where 30% of people reported perceived swelling of the knee in the absence of any objective swelling [46]) and complex regional pain syndrome (>50% report disturbances in body perception of the affected region [47,48]). To the authors’ knowledge, this is the first time that the concept of altered body perception in individuals with stroke has been explored in relation to chronic pain. Our finding is suggestive that individuals with stroke experience altered body percept at similar rates than non-stroke individuals with chronic pain conditions based on the existing literature.

Stroke survivors are considered to be at risk of experiencing body perception disturbance post-stroke [37,38]. This is viewed as likely due to somatosensory impairments that commonly occur as a result of stroke which can result in mislocalisation of tactile stimuli and reduction or proprioceptive acuity [38,41]. However, in addition to having an accurate anatomic representation of the body formed by continual processing of somatosensory information, spatial factors can also contribute to the individuals’ perception of their physical self. In people who experience spatial neglect following their stroke, there is often a failure to attend to both visual and tactile stimuli that occur in the affected portion of space [49]. This is suggestive that individuals’ body representation may also be influenced by other regions of the brain related to spatial perception affected by damage caused by the stroke.

Accurate perception of hand size is integral to effective use of the hand in its interaction with, and assessment of the external environment, most notably when holding and manipulating objects. The perceived size of body parts influences the perception of metric properties such as size and shape of objects that come into contact with the skin [50], given the measure of objects is performed with reference to perceived distance of skin of the body part in contact. It is important that the hand is perceived as a constant size in order that it can then serve as a reliable metric to enable the measuring of objects with which it interacts [51]. Experiencing ongoing hand pain is likely to result in decreased functional use of the hand and interaction with other physical stimuli, in addition to reduced movement through space, in an attempt to avoid pain. If the frequency of performance of motor activities and interactions with other physical stimuli is reduced, it is conceivable that the performance of such tasks, and the processing of environmental stimuli could become less efficient and less precise [52]. This may contribute further to alterations in perceived self and help explain the observed increase in frequency of perceived altered hand size in those who experience hand pain.

Individual participant characteristics may impact presence of chronic pain and altered body perception. More females than males identified as having chronic pain. This finding is unsurprising in a study population of individuals with stroke, given that greater prevalence of females experiencing chronic pain is commonly reported in many chronic pain states [6,53,54]. This is thought to be due to a combination of factors including genetic, hormonal, and psychosocial, and is an area of significant ongoing research [55]. Given the nature of stroke, and the contributions of somatosensation towards body percept, it was important to consider the potential impact of hemispheric differences on body perceptual disturbance. Hemispheric differences in patterns of functional connectivity within the somatosensory network have been observed in stroke for touch discrimination [56], and body perception has been lateralized to the left hemisphere [57]. Despite this, for our sample, we did not observe an influence of hemisphere of stroke lesion on the prevalence of altered body perception. Our findings support the suggestion that individuals with chronic pain post-stroke, as in other complex pain states [32,58], also frequently experience a perceptual body disturbance that does not appear to be attributed to hemisphere of lesion.

We found greater severity of neuropathic symptoms in those whose pain included the hand than in those whose pain did not. Chronic pain post-stroke can be considered a complex condition and the mechanisms behind the development and maintenance of pain following stroke are unclear. Complexity is highlighted by the varied pain symptoms that are experienced by individuals with stroke, and indeed other neurological conditions involving pathology of the somatosensory system. Studies investigating pain symptomatology across a range of neurological conditions with chronic pain have suggested that there may be common symptomatic profiles of pain experience across a range of conditions, rather than a unique profile for the specific neurological condition [59]. These profiles may be indicative of different mechanisms involved in contributing towards the pain experience [60]. This profiling based on symptomatology has been utilized to tailor medication approaches with some success in painful diabetic neuropathy [61]. Our findings of altered body image and symptomatic differences in those with and without hand pain may also be indicative of potentially different contributing mechanisms in pain presentation. These findings warrant further exploration.

Individuals with CRPS are known to frequently experience body perception disturbance mainly affecting their symptomatic limb [20,62], however we found altered body perception in 43% of those with chronic hand pain despite only 27% having been diagnosed with CRPS. This is suggestive that features commonly associated with CRPS, such as alterations in body perception, may be present across the continuum of the chronic hand pain experience in individuals with stroke, regardless of whether all criteria for diagnosis of CRPS are met. This is also the case in musculoskeletal pain—people with back pain [22] and knee pain associated with arthritis [63,64] show body perception disturbances that relate to pain, although the magnitude of the disturbance is less pronounced than those observed here or in people with non-stroke CRPS. It is possible that body perception targeted interventions developed for non-stroke CRPS may also be appropriate for individuals with post-stroke pain and body perception disturbance.

This current study of individuals with subacute and chronic stroke (N = 523) investigated perceived changes in body perception, while also looking at the symptomatic profile of the individual with chronic pain. We explored body perception localized to one region (i.e., the hand) for several reasons: the high incidence of upper limb pain [9,65,66], the problem of CRPS of the hand post-stroke [67] and the extensive coverage of body perception disturbance in non-stroke CRPS [32]. To obtain a large sample, we needed to balance detail with participant burden, so we used simple, user-friendly questions to access body perception disturbance. Formal measures using questionnaires for body percept do exist, such as the Bath CRPS Body Perception Disturbance Scale [62], the Fremantle Back and Knee Awareness Questionnaires [63,68] or versions of the Body Perception Questionnaire [69,70]. These involve multiple questions that are designed either for specific conditions [62,63,68] or include questions assessing other functions such as respiratory or gastrointestinal and were therefore considered not suitable for use in this exploratory study. The Body Perception Questionnaire includes one statement relating to perceived body shape/size: “a swelling of my body or parts of my body” [70], however we aimed to further investigate if any perceived body changes were experienced specifically to the participants’ hand in either direction, bigger or smaller because (i) non-stroke CRPS is associated with large changes in perceived hand size [19], and (ii) the perception disturbance of the hand has clear functional implications.

### 4.1. Strengths and Limitations

This exploratory study was conducted to investigate if a relationship may exist between altered body percept and chronic pain in individuals with stroke. To the authors’ knowledge, this has not been previously investigated. The aim was to first identify if a relationship exists, and to then act as a means of identifying the potential for interventions to target altered body percept if present, in the search to provide effective pain treatments. In achieving high participation by individuals with stroke, it is well-powered for these exploratory research questions. In utilizing a combination of outcome measures (NRS and the NPSI) it is consistent with the current recommendations of the Neuropathic Pain Special Interest Group of the International Association for the Study of Pain, who recommend that for studies that are trying to identify responder profiles to interventions, that a combination of a unidimensional measure (NRS or Visual Analogue Scale) and a validated neuropathic pain quality measure (either the NPSI or the Neuropathic Pain Scale [71]) are recommended [72].

We acknowledge several limitations in this exploratory study. To determine the hemisphere of stroke lesion, the survey did not utilise any diagnostic investigational data, rather side of stroke lesion was determined via a survey question to the participant. While the question did ask “What side/s of your brain were affected by your stroke/s?” and participants were presented with options of right/left/both/unknown, participants may have indicated the side of their symptoms rather than lesion side. Further, 71 participants did not answer this question. We also did not attempt to categorize pain symptomatology into different types of pain, as has been done in other prevalence studies of pain post-stroke [11,73]. Conditions such as central post-stroke pain (CPSP) lack clear diagnostic criteria [74] and due to the varied presentations of pain experiences post-stroke it can be considered that individuals experience a combination of several pain types [73,74]. Survivors of stroke often develop novel pain post-stroke [10] which then becomes chronic, and conditions such as post-stroke shoulder pain and CPSP often develop in the weeks and months following stroke [74,75,76]. This suggests that there may be adaptive contributions towards the chronic pain experience in stroke survivors, and thus post-stroke pain may be considered to have characteristics that are a mix of neuropathic, nociceptive and nociplastic, as defined by the International Association for the Study of Pain [13]. In line with current recommendations [72] for conditions where neuropathic pain characteristics are considered likely, assessments of pain intensity (NRS) and pain quality (NPSI) were included in this exploratory study.

This study was performed online and promoted through numerous means to facilitate optimal access for individuals with stroke. However, online studies may be inaccessible to many, and the sample therefore may not be representative of the general stroke population. To participate, individuals were required to have English language skills, adequate computer skills and internet access. It is also unlikely that individuals with aphasia participated, as post-stroke aphasia has been associated with negative use of the internet [77] and currently there is no recommended online pain intensity measure for individuals with aphasia [78]. We did not lodge a public protocol for data analysis prior to data collection for this study. Although this is now recommended practice in pain research [79], we commenced this study before this commendable shift in practice occurred. We acknowledge that failing to do this limits the transparency of our reporting.

### 4.2. Clinical Considerations

Body perception disturbances have clinical significance because they may cause distress due to feelings of a loss of self-ownership of the body part [32]. Survivors of stroke who lack positive body ownership may therefore also perceive their bodies as unfamiliar and unreliable, contributing to feelings of fragility and vulnerability. Perceived vulnerability will increase surveillance of environmental and internal signals consistent with threat. According to contemporary pain science, these mechanisms are likely to increase the likelihood of pain with normally non-painful stimuli (‘allodynia’) and the intensity of pain associated with normally painful stimuli (‘hyperalgesia’) [80].

We showed that altered body perception is frequently (34%) experienced by individuals with stroke and chronic pain, yet this common experience may be overlooked clinically because individuals are reluctant to report it and health professionals do not know to ask. For example, individuals with non-stroke CRPS have reported that disturbances in body perception have been perceived as negative in discussions with health professionals [20]. The perception of altered hand size is likely to be perceived as negative and worrying, and the presence of a negative body percept may drive unhelpful coping strategies [26] and contribute to increased pain [81]. People with stroke who experience chronic pain have been shown to hold rigid negative beliefs with regard to their pain experience [82]. If health professionals were to ask about body perception disturbances including any perceived size differences in body regions, while informing stroke survivors of the frequency in its occurrence, it may assist in: reassuring the individual with stroke about their experience; help the individual regain a positive body attitude [83]; and, through a reduction in stress, potentially contribute to a decrease in the individual’s pain experience.

Our new findings on the presentation of body perception disturbance in stroke may be beneficial in the identification and targeting of underlying mechanisms contributing to the pain experience after stroke, and subsequent development of effective targeted therapies and rehabilitation [20]. It has been proposed that use of interventions, such as visual illusion and imagery, targeting the individuals’ body ownership and improve representation of the upper limb may be beneficial if applied prior to conventional motor rehabilitation training in stroke [84]. What is more, a strong feeling of body ownership may well contribute to successful restoration of motor function [85]. That body perception disturbance is more common in those who experience pain post-stroke may indicate that such a relationship could be particularly relevant for stroke survivors with chronic pain.

## 5. Conclusions

We found that presence of altered body perception of hand size was more common in individuals with stroke who experienced chronic pain than it was in those who did not. Changes in body perception were further highlighted when the region of pain included the hand. This new finding contributes to the growing understanding of chronic pain in stroke and provides clinicians with insights into the relationship that exists in individuals with stroke between pain and body perception. It is hoped that this new knowledge will contribute to early identification and exploration of existing treatment strategies targeting body perception. The current findings raise the possibility that such treatments will improve the function and pain-related outcomes of a group that is currently highly impacted by pain.

## Figures and Tables

**Table 1:** Comparison of demographic, clinical and hand size data for stroke survivors with and without reported chronic pain.

	No Pain N = 199	Pain N = 324	*p* Value
Age, years (mean, *SD*)	59(14)	58 (13)	0.486 ^a^
Gender, female	43% (86/199)	56% (182/324)	***0.004*** ^b^
Reported hemisphere of lesion Right Left Both Unknown	44% (88/199) 34% (67/199) 5% (9/199) 18% (35/199)	46% (150/324) 35% (114/324) 7% (24/324) 11% (36/324)	0.644 ^b^ 0.723 ^b^ 0.188 ^b^
Duration post-stroke, years (mean, *SD*)	7.88 (6.77)	7.14 (6.20)	0.215 ^a^
Altered perceived hand size (yes)	15% (30/199)	34% (110/324)	***0.001*** ^b^
Reported nature of size change Smaller Bigger Missing	37% (11/30) 63% (19/30) -	35% (39/110) 58% (64/110) 6% (7/110)	0.902 ^b^ 0.611 ^b^

^a^ Student *t*-test, ^b^ Chi-square test factor ‘Group’.

**Table 2:** Comparison of hemisphere of lesion and hand size for individuals with chronic pain including and excluding the hand.

	Pain (Excl Hand) N = 183	Pain (Incl Hand) N = 141	*p* Value
Hemisphere of lesion Right Left Both Missing	48% (87/183) 33% (60/183) 7% (13/183) 13% (23/183)	45% (63/141) 38% (54/141) 8% (11/141) 9% (13/141)	0.609 ^b^ 0.303 ^b^ 0.812 ^b^
Altered perceived hand size (yes)	28% (51/183)	42% (59/141)	***0.009*** ^b^
Reported nature of size change Smaller Bigger	10% (19/183) 16% (29/183)	14% (20/141) 25% (35/141)	0.297 ^b^ 0.044 ^b^

^b^ Chi-square test factor ‘Hand’.

**Table 3:** Comparison of pain measures and symptoms by region of pain (excluding or including hand pain).

Pain Scale (Mean, *SD*)	Other Pain	Hand Pain	*p* Value
Numerical Rating Scale	5.97 (1.92)	6.11 (1.81)	0.514 ^a^
Neuropathic Pain Symptom Inventory * Sup Spontaneous (Burning) ** Deep Spontaneous ** *Deep Spontaneous (Squeezing)* *Deep Spontaneous (Pressure)* Paroxysmal ** *Paroxysmal (Electric Shocks)* *Paroxysmal (Stabbing)* Evoked ** *Evoked (Brushing)* *Evoked (Pressure)* *Evoked (Cold)* Paraesthesia/Dysaesthesia ** *Paraesthesia/Dysaesthesia (Ps and Ns)* *Paraesthesia/Dysaesthesia (Tingling)*	27.8 (21.6) 3.10 (3.23) 2.76 (2.81) 1.87 (2.97) 3.65 (3.42) 2.87 (2.95) 2.29 (3.15) 3.45 (3.59) 2.43 (2.53) 1.86 (2.89) 3.49 (3.42) 1.93 (33.18) 3.09 (3.05) 3.36 (3.35) 2.83 (3.14)	42.4 (22.1) 4.37 (3.16) 4.01 (2.97) 3.53 (3.22) 4.48 (3.33) 3.43 (2.97) 3.28 (3.26) 3.59 (3.42) 3.98 (2.86) 3.76 (3.27) 4.22 (3.44) 3.96 (3.39) 5.58 (3.18) 5.39 (3.33) 5.78 (3.25)	***<0.001*** ^a^ ***<0.001*** ^a^ ***<0.001*** ^a^ ***<0.001*** ^a^ 0.029 ^a^ 0.089 ^a^ ***0.006*** ^a^ 0.741 ^a^ ***<0.001*** ^a^ ***<0.001*** ^a^ 0.056 ^a^ ***<0.001*** ^a^ ***<0.001*** ^a^ ***<0.001*** ^a^ ***<0.001*** ^a^

^a^ Student *t*-test, * NPSI Total Score, ** NPSI Domain Score, Ps and Ns = Pins and Needles.

## Data Availability

The data presented in this study are available on request from the corresponding author. The data are not publicly available due to planned further analyses.

## Appendix A

1. General Information
  - Year of birth (drop down boxes 1910–1998)
  - Gender (click male/female/rather not say)
  - Country of residence (drop down boxes)
2. Have you been diagnosed by a medical practitioner as having had a stroke? (click yes/no)
  - If yes:
    (a) Was it in the past three months? (click yes/no)
    (b) How many strokes have you had (drop down boxes 1–4+)
    (c) When did you first have a stroke? (drop down boxes 1930–2018)
    (d) When did your most recent stroke happen? (drop down box 1930–2018)
    (e) Which best describes your stroke/s:
      (1) Bleed (haemorrhage)/Clot (infarct)/Both (bleed and clot)/ Not sure (drop down boxes)
      (2) What side/s of your brain were affected by your stroke/s? (click left/right/both/unknown)
3. Handedness
  - Prior to your stroke, what was your preferred or dominant hand to use? (click left/right)
  - Since your stroke, what is your preferred or dominant hand to use? (click left/right)
  - Since your stroke, does it feel like your hand is now a different size? (click yes/no)
    (a) If yes, does it feel (click bigger/smaller)?
4. Pain
  - Have you experienced ongoing pain over the past three months that has made you do something for it? (e.g., Take a tablet, change behaviours, see a health professional) (yes/no) If yes please continue
    (a) Please score your pain level out of 10 on the chart below, where 0 = no pain, and 10 = worst pain imaginable
    (b) Where? Tick more than one area if appropriate (body charts with tick boxes)
